# A scoping review of cost-effectiveness of screening and treatment for latent tubercolosis infection in migrants from high-incidence countries

**DOI:** 10.1186/s12913-015-1045-3

**Published:** 2015-09-24

**Authors:** Lorenzo Zammarchi, Gianluigi Casadei, Marianne Strohmeyer, Filippo Bartalesi, Carola Liendo, Alberto Matteelli, Maurizio Bonati, Eduardo Gotuzzo, Alessandro Bartoloni, the COHEMI project study group

**Affiliations:** Infectious Diseases Unit, Department of Experimental & Clinical Medicine, University of Florence School of Medicine, Largo Brambilla 3, 50134 Florence, Italy; Laboratory for Mother and Child Health, Department of Public Health, IRCCS – Istituto di Ricerche Farmacologiche Mario Negri, Via G. La Masa 19, 20156 Milan, Italy; SOD Malattie Infettive e Tropicali, Azienda Ospedaliero-Universitaria Careggi, Largo Brambilla 3, 50134 Florence, Italy; Instituto de Medicina Tropical “Alexander von Humboldt”, Universidad Peruana Cayetano Heredia, Barrios Altos, Lima, Peru; Institute of Infectious and Tropical Diaseases, WHO Collaborting Centre for TB Co-infection and TB Elimination, University of Brescia, Brescia, Italy

## Abstract

**Background:**

In low-incidence countries, most tuberculosis (TB) cases occur among migrants and are caused by reactivation of latent tuberculosis infection (LTBI) acquired in the country of origin. Diagnosis and treatment of LTBI are rarely implemented to reduce the burden of TB in immigrants, partly because the cost-effectiveness profile of this intervention is uncertain.

The objective of this research is to perform a review of the literature to assess the cost-effectiveness of LTBI diagnosis and treatment strategies in migrants.

**Methods:**

Scoping review of economic evaluations on LTBI screening strategies for migrants was carried out in Medline.

**Results:**

Nine studies met the inclusion criteria. LTBI screening was cost-effective according to seven studies. Findings of four studies support interferon gamma release assay as the most cost-effective test for LTBI screening in migrants. Two studies found that LTBI screening is cost-effective only if carried out in immigrants who are contacts of active TB cases.

**Discussion and Conclusions:**

Our findings support the cost-effectiveness of LTBI diagnostic and treatment strategies in migrants especially if they are focused on young subjects from high incidence countries. These strategies could represent and adjunctive and synergistic tool to achieve the ambitious aim of TB elimination.

**Electronic supplementary material:**

The online version of this article (doi:10.1186/s12913-015-1045-3) contains supplementary material, which is available to authorized users.

## Background

In low incidence countries, the majority of the cases of active tuberculosis (TB) occur among migrants from high incidence countries [1]. In this setting migrants can develop TB following three main mechanisms [2]:

1. TB can already be present at the time they enter in the host country;
2. TB can be the consequence of the reactivation of latent tuberculosis infection (LTBI) acquired in the country of origin, occurring months to years after the settlement in the host country [3, 4];
3. Primary progressive TB can follow a new infection acquired in the host country [4, 5] or during a return travel to the country of origin [6].

Most countries with low TB incidence adopt TB screening policies for migrants from high TB incidence countries. The majority of countries screen migrants for active TB through chest x-ray (CXR) before or soon after arrival, while screening for LTBI is not consistently imple mented [7]. Screening protocols that include CXR as first step are able to identify the majority of migrants with active TB at entry and, occasionally, migrants with radiological alterations suggestive of LTBI. However, most persons with LTBI go undetected as a diagnostic test for this condition is not usually applied. Epidemiological studies based on molecular techniques to genotype the *M. tuberculosis* isolates showed that 55–90 % of TB cases diagnosed in foreign born patients are due to LTBI reactivation [8, 9].

Screening migrants for LTBI and providing treatment to those with this condition is a plausible strategy to prevent the disease and reduce the risk of spread infection in the native population [10, 11]. Diagnosis and treatment of LTBI was recommended in Europe already more than ten years ago [12, 13], and is now included among the main interventions of the new global post-2015 strategy for TB control [13].

Only 16 of 29 industrialized countries belonging to the Organization for Economic Co-operation and Development, screen immigrants for LTBI, most frequently post-arrival in the host country [7].

In 11 of these 16 countries, the screening is compulsory for legal migrants. Children and young adults (<40 years) are most commonly targeted for LTBI screening. The most common test used for screening is Tuberculin Skin Test (TST), used in 11 out of 16 countries. Patients and physicians compliance to the LTBI screening protocol is essential for the effectiveness [10], but it is reported to be low [11].

Appropriate information on cost-effectiveness of LTBI screening strategies may help the policy makers to decide appropriate interventions. Thus, we performed a review of published economic evaluations (EE) of different LTBI screening strategies.

## Methods

Ethical approval was not required for this review study.

### Inclusion criteria

In this review we included studies with all the three following criteria: 1) had migrants as study target population; 2) included diagnosis and treatment for LTBI; 3) reported findings of EE analyses. Both model-based EEs and those alongside clinical trials (or in combinations as well) were included.

### Search strategy

MEDLINE and the Cochrane Library electronic databases were searched for studies published up to July 2014. The terms used for the search strategy were: (latent tuberculosis OR LTBI OR “latent tuberculosis”[Mesh]) AND (screening OR Mantoux OR IGRA OR “mass screening”[Mesh] OR “tuberculin test”[Mesh] OR “Interferon-gamma Release Tests”[Mesh]) AND (cost-effect* OR cost-bene* OR “Cost- Benefit Analysis”[Mesh]). No language restriction was done. The search was performed also using the term “latent tuberculosis screening in migrants” as free text.

### Data extraction and assessment

All references retrieved were collected using the EndNote®, version X5 (Thomson Reuter) program. Identified titles and abstracts were screened for their eligibility for inclusion and the full text of potentially relevant studies was obtained and examined. Two review authors (LZ, GC) independently screened titles and abstracts of each study. Based on the full text revision, the two reviewers independently selected the studies, and inter-reviewer disagreement was solved by discussion. The following information was extracted: setting, study design, participants, EEs data, including type of EE, screening alternatives, cost description, analysis perspective, source of data (literature, clinical studies), modeling (if any, including time horizon and discount rate), key results and authors’ conclusions. The following data were collected for clinical trials used for EE analyses: inclusion criteria, study participants, setting, design and methods, results and authors’ conclusions. Agreement on inclusion was calculated using the Kappa statistics.

Study quality was assessed using an established checklist of criteria for assessing the quality of economic evaluations in health care [14]. This tool includes ten items regarding cost effectiveness analysis: the presence of a clearly stated hypothesis and comparator; the used methods; the medical evidence; appropriate costs and benefits considered; a marginal analysis and a sensitivity analysis have been undertaken; the analysis was appropriate to the local environment.

## Results

### Search results

**Fig. 1:**
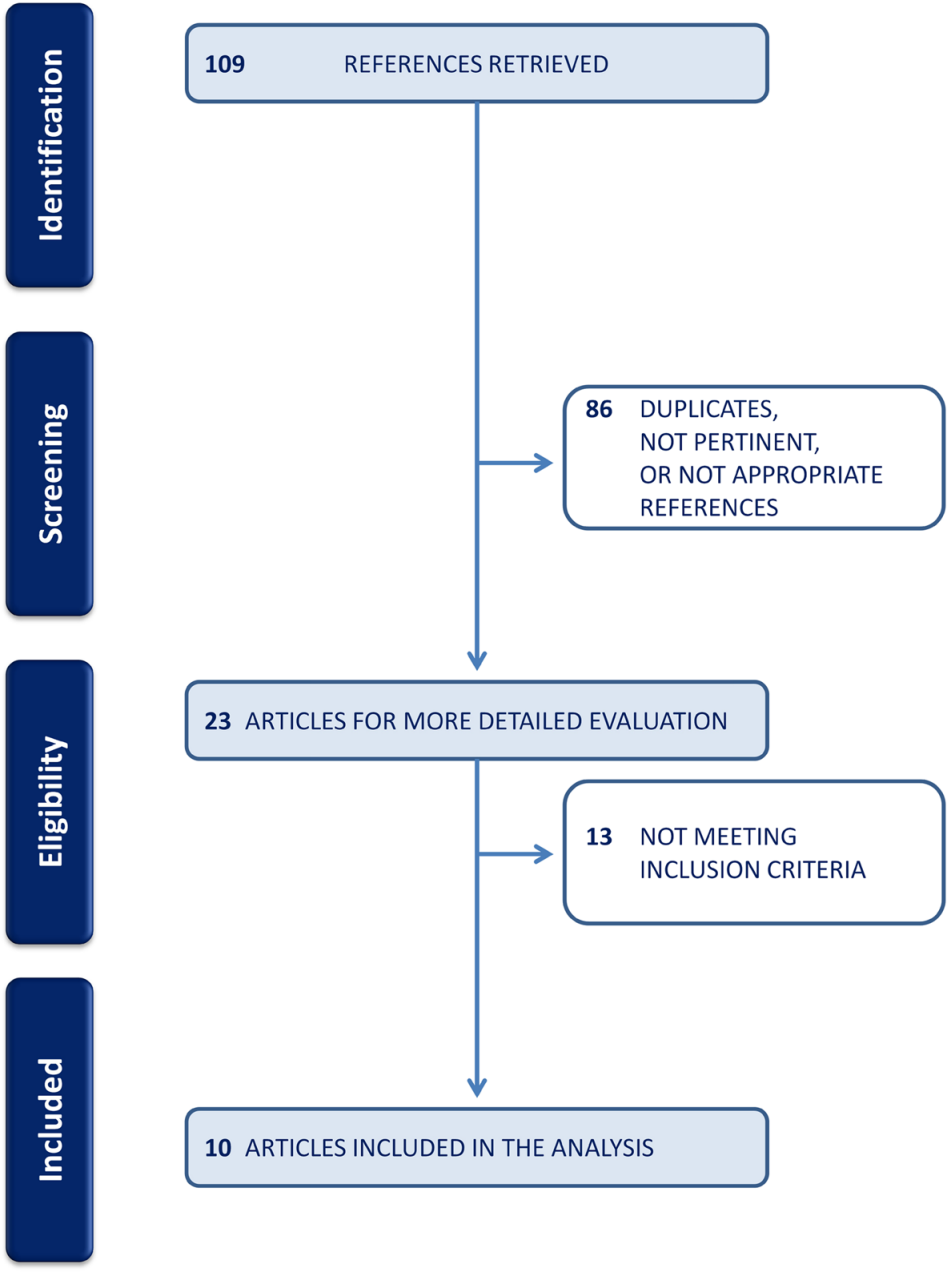
Flow diagram showing the number of papers identified by the search and the selection process

The literature search resulted in 109 titles. A total of 86 duplicates or non-pertinent or non-appropriate references were deleted, resulting in 23 potentially relevant studies. Reviewers agreed on 18 of 23 papers (78.3 %) selected for reliability check (K = 0.697), and disagreements were resolved by consensus. Ten of 23 papers (43.5 %) met inclusion criteria and were therefore included in the final step of review (Fig. 1).

### General characteristics of included studies

The 10 research studies included in the review (Table 1) were published in 8 journals, 2 in the Am J Respir Crit Care Med and Thorax, and the others in 6 different journals. The distribution of papers per year of publication ranged from 2000 to 2014.

**Table 1:** Economic evaluations of latent tuberculosis infection screenings published up to July 2014

Author (Country; Year)	Alternatives	Data source	EE Type	Perspective	Model Horizon Discount rate	Cost	Cost description	Differential Costs	ICER	Conclusion
Dasgupta et al. (Canada; 2000) [19]	1. Screening (medical evaluation, CXR);	Administrative data bases	CEA	Health care payer	Markov 20 years	Direct	CXR, clinic visits, investigations, hospitalization, drugs, physicians’ and pharmacists’ fees, administrative activities.	• TB case detected: CAN$ 31,418; prevented: CAN$ 73,125;		Close-contact investigation was highly cost-effective and resulted in net savings. Immigrant applicant screening and surveillance programs had a significant impact but were much less cost-effective, in large part because of substantial operational problems. Radiographic screening of newly arriving foreign-born populations for TB could be cost-effective and have considerable individual and public health benefits.
								• CAN$ 55,728; CAN$ 155,729;		
	2. Surveillance of inactive TB, including LTBI treatment;									
								• CAN$ 10,275; CAN$ 29,668.		
	3. Close-contact investigation									
Khan et al. (USA; 2002) [18]	1. No LTBI screening;	Data bases	CEA, CUA	Societal	Markov Lifetime 3.00%	Health Direct	Transportation, ambulatory care, services of interpreters, laboratory tests, medications, adverse drug reactions, hospitalization, and patients’ time.	Total savings: US$60 to US$90 million, assuming to avert 9–10 thousand TB infections per year.	CEA	Results variable according to country of origin.
									• TST followed by INH: savings or dominated, depending on regions.	
	2. TST followed by INH;					Indirect				A strategy of detecting and treating LTBI was cost-saving among immigrants from Mexico, Haiti, sub-Saharan Africa, South Asia, and developing nations in East Asia and the Pacific.
									• TST followed RIF: dominant.	
	4. TST followed by RIF plus PZM.								• TST followed RIF plus PZM: saving or cost/effective (US$2,551 - US$149,978 per future case averted), depending on regions.	
	3. TST followed by RIF;									
									per QALY	Screening was highly cost-effective among immigrants from other developing nations. RIF-PZM was the preferred treatment for treating LTBI in immigrants from Vietnam, Haiti, and the Philippines.
									• TST followed by INH: savings, US$914 - US$5,952, or dominated depending on regions.	
									• TST followed by RIF: dominant.	
									• TST followed by RIF plus PZM: savings, or US$1,276 - US$53,388, depending on regions.	
Brassard et al. (Canada; 2006) [17]	1. LTBI school-screening program in newly arrived immigrant children (TST followed by INH);	Clinical trial^c^	CBA	Health care payer	No	Health Direct	Total material and labor costs associated with the school-screening program and the associate investigations for children and associates.	Total savings: CAN$ 363,923.		The school-based LTBI-screening program was found to be cost-effective.
								Without associate investigation: CAN$ 268,393.		
										Savings were mainly due to hospitalization costs.
	2. Active TB management through passive case finding.									
Porco et al. (USA; 2006) [20]	1. Follow-up of TB-notification patients, including LTBI treatment for latently infected individuals;	Published literature, administrative data bases	CEA, CUA	Health care payer	Markov 20 years 3.00%	Direct	Diagnostic tests, nursing assessments and doctor visits, drugs, side effects, hospitalizations.	The program yielded 7.7 net QALYs, US$ 25,000 in net savings, and prevented 4 cases of TB.	Treatment of TB4s was cost-saving.	Domestic follow up is highly cost-effective as early detection and treatment reduces the rate of hospitalization.
									Treatment of TB2s was highly cost-effective: US$4,400 per QALY and US$4,700 per case prevented.	
	2. No follow-up.									
Oxlade O et al. (Canada;2007) [11]	1. CXR;	Published literature, administrative data bases	CEA	Societal	Markov	Health Direct	All government and health system costs, patients’ out-of-pocket expenditures, but not TB-related death or disability.	Savings only in high-very high risk populations:	CXR - the least costly ICER per case prevented: CAN$ 875 for immigrants from high-incidence TB up to CAN$ 2.2 million from low incidence.	Screening with CXR would be the most and QFT the least cost-effective.
	2. TST;				20 years					
	3. QFT;				3.00%					
	4. TST+QTF;							• CXR - CAN$ 44,710; CAN$ 65,490;		Screening for LTBI, with TST or QFT, is cost-effective only if the risk of disease is high. The most cost-effective use of QFT is to test TST-positive persons.
	5. No screening.								QFT - the most expensive: CAN$62,643 up to CAN$1,122,200.	
								• TST - CAN$ 136260; CAN$ 476320;		
									TST - better than QFT with saving up to CAN$35,000 compared to QFT, but in populations BCG-vaccinated after infancy, where TST more expensive because of low specificity.	
	Three scenarios:							• QFT - CAN$ 100,490; CAN$ 440,550.		
										Screening with TST or QFT was much more cost-effective in contacts than entering immigrants.
	a) immigration entry screening;									
										However, the selection of screening strategy is less important than program performance. Programs considering these new *ex vivo* tests for LTBI should thus first ensure that a high proportion of those with positive tests will be medically evaluated, prescribed and complete therapy.
	b) close or									
	c) casual contacts.									
Hardy et al. (UK; 2010) [21]	1. NICE guidance 2006;	Clinical trial^c^	CEA	Health care payer	No	Health Direct	Cost per case LTBI identified	Cost per case identified:		QFT-first protocol can be carried out more cheaply than a CXR-first protocol, with a cost-saving of about 35% (£67.65) compared to NICE protocol. This saving is due to the reduced number of CXRs required.
								• NICE protocol: £160.81;		
								• Leed protocol: £93.16.		
	2. Leeds protocol: QTF first in immigrants from countries with TB incidence >200/10^5^ followed by CXR (all ages, but mean age was 30.8 year).									
Linas et al. (USA; 2011) [23]	1. TST;	Published literature	CEA, CUA	Health care payer	Markov Lifetime 3.00%	Health Direct	Nursing and physician visits, diagnostic tests, medications, hospitalizations, contact tracing, and directly observed therapy.		• Individuals at highest risk of TB reactivation (close contacts and HIV-infected) - ICER of IGRA compared to TST was <$100,000/QALY gained.	In foreign-born subjects IGRA was cost-saving compared to TST and cost-effective compared to no screening.
	2. IGRA;									
	3. No LTBI screening.									
	Risk-groups^a^									
									• The foreign-born - IGRA was cost-saving compared to TST and cost-effective compared to no screening (ICER <$100,000/ QALY gained).	
									• Vulnerable populations (homeless, drug user, former prisoner) - ICER of TST screening was approximately $100,000-$150,000/QALY gained, but IGRA was not cost-effective.	
									• Medical co-morbidities (diabetes and others) - ICER of screening with TST or IGRA was >$100,000/QALY.	
Pareek et al. (UK; 2011) [15]	1. QFT;	Clinical trial^c^	CEA	Government health care payer	Markov 20 years 3.50%	Direct	UK NICE TB guidelines	Screening of immigrants from any countries irrespective of tuberculosis incidence would cost:	• Screen immigrants aged 16–35 years from countries with incidences/10^5^	Screening for latent infection can be implemented cost-effectively at a level of incidence that identifies most immigrants with latent tuberculosis, thereby preventing substantial numbers of future cases of active tuberculosis.
	2. NICE guidance 2006^b^.									
								• QFT more than £1.5 million and prevent 44.5 cases of tuberculosis;	≥250/10^5^: £ 17,956 per case averted; ≥ 150/10^5^: £ 20,819; ≥40/10^5^: £ 29,403.	
									Screen immigrants aged ≤35 years irrespective of TB incidence: £ 101,938.	
								• NICE guidance ≈£850,000 and prevent 13.2 cases.		
Pareek et al. (UK; 2012) [16]	1. TST+ in <35y old immigrants;	Clinical trial^c^	CEA	Government health care payer	Markov 20 years	Direct		Current UK national guidance associated with additional costs of between £594,957 and £1,530,303 over 20 years.	• QFN (single): £21,565 - £34,754 per TB case averted.	Mandatory CXR on arrival could be safely eliminated in order to improve screening cost-effectiveness with single-step QTF at incidence threshold >250/10^5^ per year.
									• CXR plus single QFN: £59,489.	
	2. QTF+ in <35y old immigrants;								• CXR plus single T.SPOT.TB: £402,422.	
	3. T-SPOT+ in <35y old immigrants.									
	All with or without CXR port of entry.									
Iqbal et al. (USA, 2014) [22]	1. QFT;	Administrative data base (2007)	CEA	Government health care payer	Decision model	Health Direct	Screening, CXR, Treatments, lab tests and diagnostics, physicians’ and staff time.	Total screening cost: TST US$38; QFT-G US$74	Differential costs for screening and follow-up for subjects who were estimated to be test positive on 1,000 latent TB infections	QFT is cost-effective especially for high-risk populations such as foreign-born individuals.
	2. TST.									
								Key assumption.		
								False positive rates:	• U.S. born	
								• U.S. born	QFT: +US$25,037 vs. TST.	
								TST 66%; QFT 40%	• Foreign born	
								Treatment duration: 9 months	QFT: −US$135,946.	
								• Foreign born		
								TST 69%; QFT 18%		
								Treatment duration: 9 months		

*EE* economic evaluation, *ICER* Incremental cost-effectiveness ratio, *CEA* cost-effectiveness analysis, *CXR* chest X-ray, *CAN$* Canadian dollar, *INH* isoniazid, *RIF* rifampicin, *PZM* pyrazinamide, *CUA* cost-utility analysis, *US$* United States dollar, *TST* tuberculin skin test, *QTF* Quantiferon, *QALY* Quality Adjusted Life Years, *CBA* cost-benefit analysis, *LTBI* latent tuberculosis infection, *TB* tuberculosis, *NICE* National Institute for Health and Care Excellence, TB2 subjects with evidence of infection but no evidence of disease, TB4 subjects with stable radiographic abnormalities suggestive of TB together with evidence of TB infection and negative bacteriologic studies

a) Risk groups including recent immigrant adults and children, foreign-born residents living in the U.S. for more than five years (stratified by age), close contact adults and children, HIV-infected individuals, the homeless, injection drug users, former prisoners, gastrectomy patients, underweight patients, and persons with silicosis, diabetes, and end-stage renal disease.

b) CXR in all immigrants from countries with TB incidence>40/10^5^ and >16yo; TST if <16yo or <35yo from Sub Saharan Africa or from countries >500/10^5;^ QTF in TST positive to confirm LTBI.

c) Clinical trial description reported as Additional file 1: Table S2

Four studies were performed in the US, 3 in the United Kingdom, and 3 in Canada.

In four studies age limitations in the study population were used: children 4 to 18 years old [17], migrants >16 year old (median 29 years) [16], migrants or foreign born subjects > 18 year old [18], and migrants aging <35 years [15].

Four studies considered immigrants at the moment of their entrance or application for residence permission [11, 18–20]. Two studies referred to “new immigrants” or foreign born subjects without further specification [17, 21, 22]. Two studies referred to immigrants arrived within 5 years [7, 15]. One study considered both recent migrants and foreign born residents in US for more than 5 years [23].

In 5 studies the screening was carried out according to TB incidence in the country of origin regardless of age [11, 17, 19, 20, 23], whereas 3 studies compared the cost-effectiveness of screening migrants with different thresholds of TB incidence in the country of origin [14, 16, 21]. One study considered migrants from all developing countries [18].

Several LTBI diagnostic strategies (screening with CXR and follow-up of inactive TB, screening with TST, screening with TST followed by confirmation with Interferon gamma release assay - IGRA, screening with single step IGRA) were compared with each other and/or with different non-LTBI screening strategies including no screening, screening for active TB with CXR, and close-contact investigation (Table 1). Isoniazid for 6–9 months was the LTBI treatment regimen used in the majority of studies [11, 17, 19, 20, 22, 23], in one study isoniazid plus rifampicin for 3 months was used [16], in one study either isoniazid for 6 months or isoniazid plus rifampicin for 3 months were used [15], and in one study different LTBI treatment regimens (isoniazid for 9 months, rifampicin for 4 months, rifampicin plus pyrazinamide for 2 months) were compared [18]. In one study the LTBI treatment regimen was not specified [21].

All evaluations complied with the following quality criteria: presence of a clear hypothesis, methodology, selection of comparators, choice of appropriate benefits, and local applicability. Only four evaluations referred to clinical trials [15–17, 21], while medical evidence was derived from published literature in the remaining 6 studies. Appropriate direct and indirect health costs were considered in 8 assessments and sensitivity analysis was performed in seven evaluations. The lower quality study was the oldest [19]. Additional file 1: Table S1 presents different assumptions concerning progression rate and sensitivity as well as specificity of the TST and IGRAs applied in the different papers.

### Economic evaluations of included studies

Cost-effectiveness analyses (CEA) were conducted in all EEs, and only one cost-benefit analysis (CBA) [16]. Cost-utility analysis (CUA) was performed in addition to CEA in three EEs [18, 20, 23]. EEs were conducted under the perspective of public health payer. Direct health costs were assessed in all studies while indirect costs were considered in one study [18]. As shown in Additional file 1: Table S2, four EEs were based on the results of specific studies while the remaining 6 assessments were based on available data in literature. Three studies [17, 20, 23] were carried out prospectively for 12 to 31 months. Decision modeling was adopted in 8 EEs with a time horizon up to 20 years [11, 15, 16, 19, 20] or lifetime [18, 23]. Data were collected from studies carried out *ad hoc* in two EEs [20], while models were fed with data publicly available in the remaining 6 EEs.

Screening for LTBI in migrants was cost-effective according to 8 studies [15–18, 20–23] and cost-saving according the selected scenarios in 3 of these studies [17, 18, 20]. In 5 out of 8 studies in which LTBI screening of migrants was cost-effective, a comparison between single step IGRA and TST or TST plus confirmatory IGRA was carried out [15, 16, 22–24]. In all 5 studies one step IGRA was the most cost-effective strategy (Quantiferon®, QTF in 4 studies [15, 16, 21, 22] and an unspecified IGRA in the remaining study [23]). In the 2 studies in which LTBI screening strategy was not cost-effective (dominated), the dominant strategies were contact tracing investigations in one case [17], and screening for active TB with CXR in the other case [11].

One article found that screening for LTBI with a single step IGRA was cost-effective regardless of time since immigration [23], while in other studies the cost-effectiveness was evaluated in recent migrants only.

One study compared the cost-effectiveness of screening stratified by age and showed that screening was more cost-effective when addressing recent immigrant adults than children and that screening of US foreign-born residents aged less than 45 years was more cost-effective than screening of foreign-born residents aged 45–64 years [23]. In this study patient age affected cost-effectiveness results through its impact on the lifetime risk of reactivation. In 3 other studies, in which LTBI screening was cost-effective, the analysis was limited to children [17], or young adults [15, 16].

Three studies compared the cost-effectiveness of screening at different thresholds of TB incidence in the country of origin. According to one study, the highest cost-effectiveness was reached by screening immigrants from countries with TB incidence above 200/100,000 while for two other studies, the threshold was 250/100,000 [15, 16, 21].

One article provided information on which LTBI treatment regimen (among isoniazid for 9 months, rifampicin for 4 months or rifampicin plus pyrazinamide for 2 months) would be more cost-effective according to migrants country of origin showing that pyrazinamide plus rifampicin for 2 months would be the most cost-effective treatment for migrants from Vietnam, Philippines, and Haiti, while isoniazid would be the most advantageous regimen for other migrants [18].

## Discussion

According to the majority of studies included in this review, at least three important elements can be underlined. First, screening programs for detecting and treating LTBI in immigrants lead to substantial health and economic benefits under a societal perspective. Second, a one-step IGRA-protocol is the most cost-effective strategy for LTBI screening in migrants. Third, targeting young migrants from countries at higher incidence of TB increases the cost-effectiveness of screening.

Limitations and possible biases should be considered. First, our review, even if performed in a systematic methodology, did not respect all the criteria for systematic reviews (for example we consulted only two electronic database) so we cannot make strong and quantitative conclusions. Secondly, we don’t know whether the LTBI screening strategy that seems more cost-effective according to the majority of the selected studies (the use of IGRA) would be cost-effective in all context/jurisdiction, as this heavily depends on the country-specific treatment procedures and costs. Furthermore, we don’t have the quality or quantitative weights of each study to be able to make a pooled conclusion on cost-effectiveness. All these issues can potentially be solved in a meta-analysis, that is not attempted in this review given the high inhomogeneities among the different studies included.

Additionally, all the selected studies included in the review were carried out in countries were a CXR at entry is currently performed for active TB screening of newly arrived migrants. Most of the papers aimed at defining cost-effectiveness of adding screening for LTBI to the ongoing CXR-based screening for active TB [15, 18–21, 23]. The cost-effectiveness of LTBI screening of migrants population should also be evaluated in other low endemic countries.

While most of the studies found that screening programs for detecting and treating LTBI lead to substantial health and economic benefits under a societal perspective, other studies reached opposite conclusions. The importance of how methodological differences affect results of EEs has been recently demonstrated in a systematic review on methodological aspects of cost-effective analysis of IGRAs for the diagnosis of LTBI [24]. According to this study, some of the most relevant contributing factors generating different conclusions are the study inputs selection, the inconsistencies in the costing approach, the utility of the QALY (Quality Adjusted Life Year) as the effectiveness outcome, and the manner in which authors choose to present and interpret study results.

Among the studies selected in our review, Oxlade O et al. state that screening for active TB with CXR would be the most cost-effective and QFT the least cost-effective for screening of migrants on arrival [11], while Pareek M et al. concluded that mandatory CXR on arrival could be safely eliminated in order to improve screening cost-effectiveness with single-step IGRA [16]. Opposite conclusions were reached because Oxlade O et al. assumed a very low prevalence of LTBI in arriving immigrants (0.08–2.1 %) [11], while Pareek M et al. assumed a high rate of LTBI treatment completion (85 % in case base scenario) [16].

Studies performed under program conditions have shown that completion of LTBI treatment could be much lower that what was assumed in the study published by Pareek M et al. [16] with a better trend observed when shorter and unsupervised regimen are used [25]. It will be interesting to evaluate the completion rate and cost-effectiveness of shorter regimen such as weekly-administered rifapentine plus isoniazid for three month [26, 27] in migrants.

In migrants the diagnostic delay in cases of active TB is mainly related to the delayed presentation of the patients to the health system [28, 29] and it is responsible for the spread of the disease among other members of their community. Contact investigation should therefore be strengthened in migrants, as well as in the general population [30]. However, as assumed by the majority of economic models reviewed, screening and treatment for LTBI in migrants would prevent active TB cases and solve the problem at its roots.

The reliability of Markov models, where time horizons of 20 years or life time were used, may be matter of concern. In fact, in most cases, modeling was based on published or retrospective data while the prospective trials supporting three EEs were all open label and only one was controlled. The actual epidemiological data shows that the global TB burden is reducing, though at a slow pace, at global level [31], and in industrialized countries, particularly among native populations [32]. In this scenario, while the yield of the screening for LTBI will decrease over time, however the contribution to the reduction of incidence by diagnosis and treatment of LTBI will progressively increase.

Growing consensus indicates that progress in TB control in the low- and middle-income world will require not only investment in strengthening TB control programs, diagnostics, and treatment but also action on the social determinants of the disease [33]. To reduce the incidence of TB, the drivers of the epidemic and social determinants of TB need to be addressed. These include co-morbidities, substance use, the social and economic conditions that determine both the course of the TB epidemic and exposure to these risk factors [34]. This is probably true for control of TB in migrants that often live in disadvantaged socio-economic conditions in the host country with an increased risk of both, to reactivate LTBI or to acquire a new infection. In this perspective LTBI screening and treatment in migrants could represent a synergistic tool to achieve the ambitious aim of TB elimination.

## Conclusions

The majority of the studies support the use of LTBI screening strategies in migrants based on their cost-effectiveness findings. When LTBI screening for migrants are implemented they should focus especially on young migrants from high incidence countries and effort should be done to maximize the adherence to LTBI treatment. In this view shorter LTBI treatment regimen are preferred. Based on our review of EE analysis studies, the use of one step IGRA is the best option in this particular setting. These findings should be confirmed by a cost-effectiveness evaluation based on a medium-term prospective study.

LTBI should be well integrated among the TB control program for migrants and must be part of a wider approach with the aim of facilitating access of migrants to the national health system, re-orienting health services, improving the adhesion to anti-tuberculosis treatment in cases of active TB, and promoting early diagnosis of active TB cases by primary care health operators [35].

## Additional file

Additional file 1: Table S1. Different assumptions concerning progression rate and sensitivity as well as specificity of the TST and IGRAs applied in the different papers. **Table S2.** Clinical trials referring to economic evaluations. (DOCX 22 kb)
