# Supplementary material for: A scoping review of cost-effectiveness of screening and treatment for latent tubercolosis infection in migrants from high-incidence countries
Source: BMC Health Serv Res. 2015 Sep 24;15:412. doi: 10.1186/s12913-015-1045-3 (PMC4581517; doi:10.1186/s12913-015-1045-3)
Supplement: Additional file 1: Table S1. — Different assumptions concerning progression rate and sensitivity as well as specificity of the TST and IGRAs applied in the different papers. Table S2. Clinical trials referring to economic evaluations. (DOCX 22 kb) [file 12913_2015_1045_MOESM1_ESM.docx]

**Supplementary data**

**Article title: A scoping review of cost-effectiveness of screening and treatment for latent tubercolosis infection in migrants from high-incidence countries.**

**Additional file 1: Table S1.** **Different assumptions concerning progression rate and sensitivity as well as specificity of the TST and IGRAs applied in the different papers**

| **Author**  (Country;Year) | Progression rate | Sensitivity of TST | Specificity of TST | Sensitivity of IGRA | Specificity of IGRA |
| --- | --- | --- | --- | --- | --- |
| Dasgupta et al. (Canada;2000) | Annual incidence of active TB 0.1% for subjects with LTBI with no CXR lesions  Annual incidence of active TB 0.6% for subjects with LTBI and fibronodular disease at CXR | Not used in this study | Not used in this study | Not used in this study | Not used in this study |
| Khan et al. (USA; 2002) | Estimated with the formula 1-e^-I(δ T)^ where I is the average postmigration incidence of active tuberculosis infection among immigrants with latent disease, and  δ T is the expected number of years of life after entry into the United States | 98% or 100% according to two different scenarios | 85% or 95% according to two different scenarios | Not used in this study | Not used in this study |
| Brassard et al. (Canada;2006) | 5% or 10% of subjects with LTBI will develop active TB later on life according to two different scenarios | 90% or 95% according to two different scenarios | 90% or 95% according to two different scenarios | Not used in this study | Not used in this study |
| Porco et al. (USA; 2006) | The estimation of risk of reactivation is extensively described in the Appendix 1 of the publication. The authors reported a variable risk in different categories of subjects. | 93% or 95% according to two different scenarios | 99% or 95% according to two different scenarios | Not used in this study | Not used in this study |
| Oxlade O et al. (Canada;2007) | Not available | 95% | In not BCG-vaccinated: 98%  In BCG-vaccinated in infancy: 92%  In BCG-vaccinated in older childhood/adolescence: 60% | 95% | 98% |
| Hardy et al. (UK; 2010) | Not reported | “High sensitivity” not otherwise specified | Assumption: no false negative | Not reported | Not reported |
| Linas et al. (USA; 2011) | The estimation of risk of reactivation is extensively described in the Appendix 1 of the publication. The authors reported a variable risk in different categories of subjects | 89% | 92% | 83% | 99% |
| Pareek et al. (UK; 2011) | Risk of progression over 20 years period 2.5%-15% | Not used in this study | Not used in this study | 84% | 99% |
| Pareek et al. (UK; 2012) | The estimation of risk of reactivation is extensively described in the Supplementary material of the publication | In HIV negative individuals: 77%;  In HIV positive individuals: 71%. | 78% | In HIV negative individuals: 84%;  In HIV positive individuals: 75% | 99% |
| Iqbal et al. (USA, 2013) | 10% of subjects with LTBI will develop active TB later on life | 77% | 59% | 78% | 96% |

Abbreviations:

TB= tuberculosis; CXR= chest X-ray; TST= tuberculin skin test; QTF= Quantiferon; LTBI= latent tuberculosis infection.

Additional file 1: Table S2. Clinical trials referring to economic evaluations

| Author (Year) | Population | Alternatives | Study design | Efficacy | Efficacy results | Conclusion |
| --- | --- | --- | --- | --- | --- | --- |
| Brassard et al.  (2006) | 2,524 newly arrived immigrant children (aged 4-18 years) | 1. School-based screening program 2. None | Retrospective (1998-2003)  Uncontrolled | Primary: TST-positivity rate (induration of > or =10 mm  Secondary: rate of adherence to LTBI therapy, estimation of factors associated with adherence | - 542 (21%) were TST-positive - Adequate adherence in 92% (316) of 342 children started on therapy - 211/555 associates found TST positive (31%) | School-based screening program effective. Extra benefit given by adding associates to such a program. |
| Hardy et al.  (2007)  related to Hardy et al. (2010) | 280 immigrants from countries with TB incidence >200/10E5 | 1. QFT as first screening and CXR if positive 2. None | Prospective  (12 months)  Open label  Uncontrolled | Detection of LTBI | QFT: 105 positive (37,1%) compared to 83 estimated with CXR+TST program | Overall, using QTF blood testing followed by CXR is more effective than NICE guideline for screening new entrants from high risk countries |
| Pareek et al.  (2011) | 1,229 young adults (aged 16-35 years) originated from Indian subcontinent and sub-Saharan Africa. | 1. Symptom questionnaire + IGRA (QTF) 2. None | Prospective  (31 months)  Open label  Uncontrolled | Detection of LTBI | 20% positive, estimated to be more effective than NICE guidance (5,9%). | Screening for LTBI is cost-effective at preventing substantial number of future cases of active tuberculosis. Assessment of cost per TB case prevented appears more reliable than cost per QALY, as objective data on QALY were still scarce. |
| Pareek et al.  (2012) | 231 Immigrants aged ≥16 years | 1. TST 2. IGRA (QTF and T-SPOT.TB) | Prospective  (21 months)  Open label  Controlled^1^ | Detection of LTBI | TST positive 30.3%;QFN: 16.6%; T-SPOT.TB: 22.5%. | TST positive significantly higher than QTF and T-SPOT.TB. Positive finding independently associated with increasing TB incidence in immigrants' countries of origin. |

Notes:

1) Both TST and IGRA performed for each subject.

Abbreviations:

CXR= chest X-ray; TST= tuberculin skin test; QTF= Quantiferon; LTBI= latent tuberculosis infection; TB= tuberculosis; NICE= [National Institute for Health and Care Excellence](https://www.google.it/url?sa=t&rct=j&q=&esrc=s&source=web&cd=1&cad=rja&sqi=2&ved=0CDAQFjAA&url=http%3A%2F%2Fwww.nice.org.uk%2F&ei=qCL8UeGTBqbU4QT8g4CQAg&usg=AFQjCNHQH5AVUSoQS-c3XYzaIBqjSZg8Qg&bvm=bv.50165853,d.bGE)
